# Development of Databases on Iodine in Foods and Dietary Supplements

**DOI:** 10.3390/nu10010100

**Published:** 2018-01-17

**Authors:** Abby G. Ershow, Sheila A. Skeaff, Joyce M. Merkel, Pamela R. Pehrsson

**Affiliations:** 1 Office of Dietary Supplements, National Institutes of Health, Bethesda, MD 20892, USA; merkelj@od.nih.gov; 2 Department of Human Nutrition, University of Otago, Dunedin 9010, New Zealand; sheila.skeaff@otago.ac.nz; 3 Nutrient Data Laboratory, US Department of Agriculture, Beltsville, MD 20705, USA; pamela.pehrsson@ars.usda.gov

**Keywords:** iodine, database, food, dietary supplements, food composition

## Abstract

Iodine is an essential micronutrient required for normal growth and neurodevelopment; thus, an adequate intake of iodine is particularly important for pregnant and lactating women, and throughout childhood. Low levels of iodine in the soil and groundwater are common in many parts of the world, often leading to diets that are low in iodine. Widespread salt iodization has eradicated severe iodine deficiency, but mild-to-moderate deficiency is still prevalent even in many developed countries. To understand patterns of iodine intake and to develop strategies for improving intake, it is important to characterize all sources of dietary iodine, and national databases on the iodine content of major dietary contributors (including foods, beverages, water, salts, and supplements) provide a key information resource. This paper discusses the importance of well-constructed databases on the iodine content of foods, beverages, and dietary supplements; the availability of iodine databases worldwide; and factors related to variability in iodine content that should be considered when developing such databases. We also describe current efforts in iodine database development in the United States, the use of iodine composition data to develop food fortification policies in New Zealand, and how iodine content databases might be used when considering the iodine intake and status of individuals and populations.

## 1. Introduction

Low levels of iodine in the soil and groundwater are common in many parts of the world, often leading to diets that are low in iodine. Severe iodine deficiency is now rare due to widespread salt iodization, but mild-to-moderate deficiency is still prevalent even in many developed countries [1]. Knowledge about all sources of dietary iodine, including foods, beverages, water, salts, and supplements, is important for understanding patterns of iodine intake and for planning interventions. Robust food composition tables specific to individual countries are a key practical resource in providing population-level and individual-level guidance for better iodine nutrition. This article will discuss the importance of well-constructed databases on the iodine content of foods and dietary supplements, the primary causes of variability in iodine content, the desirable characteristics of these databases, and their current availability worldwide. We also describe recent progress in iodine database development and use in the United States (US) and New Zealand, and consider database applications relevant to the assessment of iodine intake of populations and individuals.

## 2. Background

Iodine is essential for the synthesis of thyroid hormone and thus is required for normal physical, neurological, and intellectual growth of infants and children, and for normal metabolism and function in adults. On a body weight basis, infancy and early childhood are the times of highest iodine requirements. Pregnant and lactating women also have increased requirements to meet their heightened physiologic needs. It is critical that women who are likely to conceive, or are pregnant or lactating, have iodine reserves sufficient for their own health and also sufficient to provide the fetus and infant with the necessary iodine supply [2,3]. The most serious consequences of iodine deficiency are well characterized and include hypothyroidism, neuro-cognitive impairment, and, in cases of severe deficiency in pregnancy, cretinism in the infant. In contrast, the consequences of mild-to-moderate iodine deficiency are less well understood and are an important priority for research and public health practice. In particular, concerns center on the impact of mild-to-moderate iodine deficiency in pregnancy, which has a high prevalence worldwide [1], on child development. Two observational studies found an association between inadequate iodine status in pregnancy and poorer academic performance in their children [4,5], although a recent randomized controlled trial reported no difference in cognitive scores of children born to mildly iodine deficient mothers supplemented with iodine or placebo in pregnancy [6]. 

Satisfactory iodine nutrition can be achieved in most circumstances through intake of adequately iodized salt in sufficient quantities and/or intake of other iodine-rich foods that are commonly consumed within a country [7]. In the early 1920s, iodized table salt was introduced in many countries, a practice that since then has spread to include the majority of countries with about 86% of the world’s population recently estimated as having access to iodized salt [8]. World Health Organization (WHO) recommends Universal Salt Iodization, whereby all salt for human and animal consumption is iodized including salt used in the food industry [9]. Some countries add iodized salt to only a few foods, for example in New Zealand and Australia, where the mandatory use of iodized salt in commercial bread production was implemented in 2009. However, in other countries iodized salt may be available but not used in commercially prepared food [10,11], or the salt may be iodized but at a very low level [10]. 

Iodine deficiency has re-emerged in countries such as Australia [12] and New Zealand [13]. A drop in iodine intake may reflect recent changes in food consumption patterns in which home-prepared foods, traditionally made with iodized salt, have been replaced with commercially prepared foods made with non-iodized salt. For example, in the case of the US, this point is reinforced by surveys documenting that retail sales of iodized salt have declined [11] and that less time is now spent preparing foods at home [14]. This situation has raised concerns about potentially inadequate intakes of iodine despite high intakes of salt from commercially prepared foods [15]. Likewise, individuals or ethnic groups whose diets exclude or restrict iodine-rich food sources for health, religious, or other reasons (such as vegan/vegetarian diet patterns, lactose intolerance, or low salt diets) may be at risk for inadequate iodine intake [16]. Knowing the iodine content of available foods thus becomes a key component in understanding which foods are the most important contributors to iodine intake for populations as well as individuals. 

The need for improved data on the iodine content of foods and beverages has been noted by several expert committees [17,18] and has recently been reviewed in detail [19]. Also, iodine derived from dietary supplements must be included along with foods in order to accurately assess total intake; therefore, data are needed on the iodine content of supplements [20]. Robust approaches to developing databases will include choosing appropriate analytical methodology (including use of standard reference materials [21,22]); designing and implementing sampling plans with good coverage of major country-specific contributors (from foods, beverages, dietary supplements, and salt); and publishing the results in database formats or tables that allow linkage with population surveys and individual intake records (such as food frequency questionnaires and 24-h recalls).

## 3. The Availability of Databases Including Iodine Content

Many countries have developed national databases that include information on the iodine content of foods, beverages, and salts, and other food components. To identify food and nutrient composition databases for individual countries and to determine if these databases included values for iodine, and also to identify information on national iodization programs for salt or other foods, we conducted an extensive Internet search using resources from FAO INFOODS [23], the recently released ILSI interactive tool [24], and other sites including Google and Google Scholar. In most cases the keywords used were “food composition”, “food composition database”, “iodine composition”, “iodine in foods”, and the name of the country using countries listed by the US Department of State. (https://www.state.gov/misc/list/). We did not specify any particular language in our search, although many databases were available in English either in their original form or in translation. 

Over 124 countries have known salt iodization programs, either mandatory or voluntary [25], but for many of these countries it was not possible to determine if the country had a national food composition database, and if so, to ascertain the presence or absence of iodine data. Table 1 presents information on the availability of national food composition databases, by country, that have iodine content data, and it also describes national iodization practices. Most databases provide for all kinds of foods with a few exceptions, such as Turkey’s database, which contains iodine values only for table salt, fish, and shellfish. However, as shown in Table 2, national iodine databases are not currently available for many countries that do have national food composition databases containing information on other nutrients. In some cases, limited iodine datasets have been published as part of scholarly or other journal publications [26,27,28,29,30,31]. (NOTE: The compilation presented in Table 1 and Table 2 is not exhaustive, nor is it equivalent to a systematic literature review. The information is dynamic, meaning that at the time of this publication, links to databases were verified to be active; however, links may change or become inactive over time.)

## 4. Sources of Variability in Food Iodine Content 

The amount of iodine in foods can be highly variable, and the nature and degree of this variability can have implications for the complexity and cost of developing databases of iodine content. For example, high variability may affect sampling plans such that more samples may need to be collected over a wider range of geographic areas and a larger number of sales or distribution venues [57]. Also, different chemical assay approaches (i.e., methods and reference materials) may be needed, depending on the anticipated range of iodine concentrations. (Note: see Section 5, below, for further discussion of these methodological issues). Highly variable and non-normal (skewed) distributions of iodine content may be best served by presentations of descriptive statistics that include multiple indicators of central tendency and range [58]. Some of the factors affecting between-country and within-country variability in iodine content of foods are described below.

### 4.1. Water 

Drinking water is particularly variable in its iodine content between and within countries, especially if they are geographically diverse. Levels of iodine in drinking water supplies are reflective of factors such as iodine in the soil and water table, proximity to sea water, and agricultural runoff [59]. Therefore, assessment of iodine intake from drinking water may require data at the regional or local level. For example, the iodine content of water in some regions of China is sufficiently high to lead to excessive intake and potential thyroid hypertrophy in school children [60]. Conversely, the desalinated water used in many parts of Israel has been noted to have very low, essentially zero, iodine levels [61]. In many countries, there is very little information available on the iodine content of drinking water supplies, nor on the iodine content of bottled waters. Food composition databases should include a range of values or a default average value for the iodine content of drinking water.

### 4.2. Salt 

Salt is the preferred vehicle for iodine fortification, and salt iodization has been implemented in 124 countries [62]. Salt can be produced from underground rock salt deposits, natural brine, or by evaporated seawater, with the latter containing <1 mg iodine (I)/kg of salt. This relatively low level of iodine in un-fortified sea salt may not always be appreciated by consumers. Most food-grade salt requires the addition of iodine, usually as potassium iodate or potassium iodide. WHO suggests that the amount of iodine added to salt should be based on the estimated salt consumed by the population; this ranges from 14 mg I/kg when estimated salt intake is high (i.e., 14 g/day) to 65 mg I/kg when salt intake is low (i.e., 3 g/day) [63]. In many countries, the iodine content of iodized salt is legislated and specified by a food standards code. However, the actual iodine content of iodized salt may differ from the reported content, particularly when iodized salt is kept in open containers and exposed to high humidity; the iodine content of salt can vary by 8% to 49% under such conditions [64]. Database developers must consider whether the country practices Universal Salt Iodization, in which case all salt for human use must be iodized, and therefore iodized salt will be used for commercial food products [9]. Alternatively, databases might include paired iodine values for some food products that have been prepared with iodized or non-iodized salt. 

### 4.3. Agricultural Practices—Soils and Crops 

Iodine occurs naturally in the earth’s crust and is present everywhere in the environment. Soils, shales, and coal rich in organic matter are generally higher in iodine than hard rock [65,66]. Also the iodine content of the soil can be influenced by the proximity of the growing area to ocean water (through which atmospheric iodine is incorporated into rainfall, and thereby raises the iodine content of the soil), the iodine content of ground waters and irrigation waters, and the use of iodine-containing fertilizers [67]. The iodine content of plant crops is affected by the content of iodine in the soil (i.e., plants grown on high iodine soils will contain more iodine than those grown on low iodine soils), but in general, plant-based foods such as vegetables and fruits are relatively poor sources of iodine [59]. The exceptions are seaweeds, which have a great capacity to concentrate iodine [68,69,70].

### 4.4. Agricultural Practices—Animal Husbandry

Dairy products and eggs may contain significant but variable amounts of iodine, influenced, to some degree, by the iodine content of supplements in animal feeds and salt licks. These supplements are often provided as part of animal husbandry practice to ensure good health and reproductive outcomes in dairy and beef cattle, sheep, goats, and poultry. Dairy products also have contained adventitious iodine from iodophors, iodine-containing disinfectants used at various points in the production of milk. Iodophors can be used to clean udders, but if the cleansing has not been performed properly some of the iodine from the teat dips can be absorbed and transferred to milk and meat. A recent US Department of Agriculture (USDA) report found that 55% of dairy operations were using iodophor teat dips [71], suggesting that the practice is still relatively common in the US. Iodophors were also used in the cleaning of industrial equipment for processing milk in dairies; a decline in this practice in New Zealand by the mid-1980s is suggested to be responsible for a drop in the iodine content of milk and dairy products [13].

### 4.5. Food Processing 

Commercial baked goods are another source of iodine when iodates are used in the commercial baking industry as dough conditioners. Iodates were introduced in the US 40 years ago and in Tasmania, Australia, over 50 years ago, but now other dough conditioners are being used. Also, some commercial baked goods contain erythrosine (Red No. 3/E127), a common food coloring that contains iodine; however, the iodine from erythrosine is only partially bioavailable [72].

## 5. Developing an Iodine Database for US Foods—Recent Progress

In 2014, the National Institutes of Health’s Office of Dietary Supplements (ODS), Bethesda MD, convened several working groups to consider clinical and population research relevant to human iodine nutrition, particularly in the US [73]. Key areas of applied and clinical research that were reviewed and identified as needing greater effort included: assessing the iodine concentration of US foods and drinking water supplies; evaluating iodine intakes of various US population subgroups; and having a sufficient scientific knowledge base to determine iodine requirements at different lifecycle stages [73]. A database on the iodine content of US foods was considered to be a critical tool for conducting research on all of the identified gap areas. This priority has been operationalized through an interagency agreement (NIH IAA-AOD-17002) between ODS and the USDA to develop the USDA Food Iodine Database. Other federal partners involved in this project include the US Food and Drug Administration and the National Institute of Standards and Technology (NIST). 

In this section, as summarized in Table 3, we will describe the main features of the project plan for the nascent USDA Food Iodine Database. A similar scientific and technical approach has been used to develop other databases, including those for choline and flavonoids [74,75]. We note that the scientific and technical approach used by the USDA is, of course, most suitable for the US; however, the concepts and operational components driving the design and execution of the project plan can serve as a prototype for development of iodine databases in other countries. 

### 5.1. The Design Phase

For any research initiative designed to provide foundational data to explore the connection between food and health, the researcher must address what and why it needs to be done, and how to achieve the answers. Put another way, when developing a new database or dataset, it is important to define the research needs and potential impact at the outset of the project. In the case of iodine, sufficient iodine composition data on food and dietary supplements are needed to estimate intakes and to assess the consequences of deficiency or excess. 

Preliminary planning activities include: identifying the “population of interest” of foods and supplements; designing the sampling plan; developing a defensible analytical process (methods and quality control); planning for statistical analysis of the results; and considering a means of data dissemination. In the case of iodine, although the USDA has developed special databases for other nutrient-focused datasets, it was important to identify unique characteristics of iodine-contributing foods and dietary supplements. Other specific challenges related to database development included the need for methods for effective sampling and analysis of foods [19], and for statistical approaches that can accommodate variability in the iodine content of foods [58].

Since resources for composition analyses are rarely sufficient to analyze all potential contributors to iodine intake, it is essential to identify existing useable data of adequate scientific quality. The USDA has long sought to take advantage of published and other available data when data quality criteria have been met to satisfaction [76]. Also, for this same reason of efficiency, when possible, the USDA seeks to harmonize its data with other data generated by complementary activities, most notably, the US Food and Drug Administration (FDA) Total Diet Study (TDS). The FDA TDS is an ongoing program that obtains about 290 foods of various matrices, four times a year, each in a different US location, sampled in order to monitor an array of nutrients and other constituents of the US food supply [77]. For iodine analysis of TDS foods, the FDA has recently synchronized their laboratory assay method, inductively coupled-mass spectrometry (ICP-MS), to that used by the USDA; in addition, the possibility of coordinating selected efforts is under discussion. This approach allows inter-agency exchange of data on iodine. Also, enhanced quality control is achieved through close collaboration to utilize NIST standard reference materials and methods. Thus, existing data from the TDS will be used to enhance the national iodine dataset and also to set priorities for planned new or repeat analyses [19]. When planning the sampling of specific foods, it is critical to identify and acquire other relevant data that clarifies the distribution of iodine in the food supply (e.g., industry uses, agricultural production, sales, and other sources). Another step involves preparing country-specific proportional weighting factors for different versions of the food; an example would the use of industry data on relative amounts sold or used of different types of iodized and non-iodized salt (e.g., conventional, sea, Kosher, etc.).

In developing the sampling plan, it is necessary to determine an appropriate sample size. The acquired food samples must be nationally representative, and the number of samples must be sufficiently large to develop statistically defensible variability estimates. The approach to this plan must be suitable for the country whose food supply is being analyzed; countries with a highly structured and nationally distributed food supply will need an approach different from that in countries where foods are acquired (grown, hunted, or foraged) and consumed within multiple smaller localities. An example of necessary adaptations is that of acquiring food samples from American Indian reservations and Alaska Native villages [78]. In the US, the sampling plan includes identification and procurement of US-representative food samples; many of these samples are selected for analysis under the NIH-USDA National Food and Nutrient Analysis Program (NFNAP) [79]. The USDA typically acquires its food samples in a minimum of 12 geographically diverse locations selected according to current population density data, retail sales data, and other national-level information. NFNAP foods under evaluation for iodine analysis include finfish and other seafood products; seaweeds/seaweed extracts; iodine-containing commercial ingredients and additives; highly-consumed dairy and egg products, commercially processed mixed dishes; retail salts; and other foods containing significant amounts of iodine. 

### 5.2. Preliminary Study Phase

It is necessary to confirm the stability of the analytes in both new and archived analytical samples, particularly when samples are shared from other studies or have been stored for some time. This is a particularly important step when analyzing for iodine in salt and other food substrates. To investigate the possible loss of iodine during sample storage, the USDA reanalyzed a set of samples that had been initially analyzed 5 years previously and was able to confirm that the iodine content of archived NFNAP foods stored at −60 °C had not changed [78,80]. The USDA also plans to evaluate salt industry data regarding iodine volatilization under varying storage, humidity, and temperature conditions.

Sample handling protocols must be developed prior to sample collection to ensure sample integrity during shipping and storage. Pilot testing of protocols including chain-of-custody plans may be warranted to ensure that sample integrity can be maintained from the point of purchase all the way through to analysis. The USDA has developed sample handling protocols for NFNAP that can serve as examples for others who are undertaking collection and eventual assay of foods [76]. An important precaution when utilizing samples of varying provenance (such as from a multicenter study) is to ensure that there has not been inadvertent contamination during the collection or storage process; sources of contamination may include leaching from storage containers that can release iodine into the sample over time and use of iodine-containing preservatives or disinfectants.

For the chemical analysis of foods, it is important to identify appropriate analytical methods and to select a capable laboratory through precertification testing with blinded samples [75]. In terms of analytical methodology, the ICP-MS method [76] is suitable for the analysis of iodine in a variety of foods, as well as dietary supplements, and is known to have good precision and accuracy. Using the ICP-MS method, the USDA contract laboratory has shown that it can generate accurate iodine values that compare well with the iodine values of several NIST-certified reference materials (CRM), notably NIST 1849a Infant/Adult Nutritional Formula and 1548a Typical Diet [21,78,80]. For quality control during analysis of samples, the USDA is using these CRM reference materials, as well as in-house control materials cross-validated to the NIST materials. Other NIST CRM materials with available iodine values (such as iodized salt, egg powder, whole milk powder, and kelp) will be used as warranted as the analytical plan progresses.

### 5.3. Research Implementation Phase

The USDA’s experience with other database development projects has revealed that the greatest effort and cost are incurred when acquiring food products according to the statistical design, conducting direct assays of the prepared samples and quality control materials, and assembling the data in an orderly format. This phase of the USDA Food Iodine Database project is now in progress. To date, over 135 unique foods have been sampled; some of these are newly acquired and others are existing frozen samples that were archived as part of previously completed projects. About 350 prepared samples representing these foods have been analyzed to date, along with quality control materials comprising about 10% of total assays. The predominant food groups analyzed so far are multi-ingredient commercial foods (e.g., restaurant hamburgers and macaroni and cheese, retail frozen pizza, milks and yogurt) and several types of fish (shellfish, crustaceans, mollusks, and finfish). 

Following data quality review, data dissemination in the form of accessible databases and professional and peer-reviewed publications will allow transparency of the data and related information. Additional ancillary studies can enhance the value and usefulness of database resources; for example, the USDA is considering a sub-study to utilize direct chemical assay followed by statistical modeling to evaluate the iodine content of home-prepared recipes in comparison with commercial food equivalents. In addition, the USDA will use ancillary studies to explore other factors that that may affect total dietary iodine such as geographic origin of foodstuffs (as reflecting iodine content of soils), the iodine content of water supplies used for drinking and reconstituting foods, and the iodine content of dietary supplements.

## 6. Iodine Content of Supplements 

Nutrient-containing supplements may contribute substantially to total nutrient intake [27,81,82], and therefore should be included along with foods when estimating total dietary intake of iodine. Supplements are used worldwide but are regulated very differently among countries [83], sometimes as foods (as in the US and Europe) [84,85], and sometimes as a form of herbal medicines (as in Germany) [86,87], and product registration or licensing may be required under certain circumstances [88]. Databases describing the content, ingredients, and other information on supplements may be assembled by government entities, manufacturers, or researchers. The information content, definitions, terminology, data formats, and sources of data can vary considerably, and this lack of uniformity and consistency has been noted as a limitation in making between-country comparisons for research and other purposes [85].

Identifying and then accessing dietary supplement databases from different countries can be challenging as there are few readily available and comprehensive compilations [85]. Nevertheless, the task may be approached on a country-by-country basis. For example, the Netherlands provides a database on the composition of supplements that is linked to the national food composition tables [89]; this database has been used as part of an iodine intake assessment study in children and adults [90]. Similarly, dietary supplement databases have been developed and then used for iodine intake assessment in pregnant and lactating women in Norway [26,27,28] and in pregnant women [29] and adults [30] in Denmark. The Australian dietary supplement database (AUSNUT 2011–2013) contains 35 nutrient values, including iodine, for 2163 dietary supplements consumed during several national nutrition and physical activity surveys conducted from 2011–2013 [91]. The method for constructing a dietary supplement database for adult participants in one of the United Kingdom sub-projects of a multi-country European cohort study has been described in detail [92]. 

In the US, the Dietary Supplement Label Database (DSLD), sponsored by ODS in collaboration with other US federal agencies, is presently the most comprehensive listing of label information (www.dsld.nlm.nih.gov/dsld-mobile/) [93,94]. The DSLD evolved from earlier questionnaire-based efforts to understand the magnitude of the contribution of supplements to population nutrient intake in the US, starting with the 1999 cycle of the National Health and Nutrition Examination Survey (NHANES) [93,95]. Other currently available databases with label information for US products include DailyMed (dailymed.nlm.nih.gov/dailymed/) and the industry-sponsored Supplement Online Wellness Library (OWL) database (www.supplementowl.org). 

Iodine content (per serving) and source (ingredients) are provided for US supplement products as listed on the product label and the Supplement Facts Panel [96]. For example, the label may indicate that the iodine in the supplement product may come from diverse sources such as iodine salts (e.g., potassium iodide) or botanical ingredients (e.g., kelp). When using label information, whether by direct inspection of the product container or through the label databases mentioned above, an important caveat is that results from direct analysis of individual supplement products usually are not publicly available. 

To estimate typical nutrient content values—including for iodine—for certain grouped classes of sampled supplement products (e.g., adult multivitamin/multiminerals (MVMs), pediatric MVMs, and non-prescription prenatal MVMs), direct chemical analysis has been conducted on sampled products and standard reference materials [97]; the analysis results have been made available through the Dietary Supplements Ingredient Database (DSID) [20,98]. The DSID assay results suggest that the actual iodine content for adult MVMs, pediatric MVMs, and non-prescription prenatal MVMs may typically exceed the labeled amount by 20–26%. Furthermore, comparisons of labeled iodine contents of prescription and non-prescription prenatal supplements sold in the US have found that, per tablet, non-prescription prenatals contain approximately 10% more iodine than prescription prenatals [99,100].

Given the concerns about the adequacy of iodine intake by pregnant women, data from the US National Health and Nutrition Examination Survey (NHANES) program has provided a means of understanding the contribution of dietary supplements to iodine intake. Use of supplements (multi-vitamins or MVMs) was found to be widespread (~75%) among US pregnant women surveyed in 1999–2006; however, use of iodine-containing supplements was relatively low (~22%) [95]. Since that time, various professional organizations including the American Academy of Pediatrics [101], the Endocrine Society [102], the Teratology Association [103], and the American Thyroid Association [104], as well as the Australian National Health and Medical Research Council [105] and the New Zealand Ministry of Health [106], have recommended that pregnant and lactating women take a daily prenatal MVM supplement that contains 150 μg of iodine. Data on time trends in usage of iodine-containing supplements by pregnant and lactating women will help in understanding whether usage is changing in response to professional recommendations. A recent study undertaken in New Zealand reported that 52% of pregnant and lactating women followed the recommendation for a 150 μg of iodine per day [107].

## 7. New Zealand: A Case Study Illustrating the Need for a Database with Iodine Content

New Zealand has low levels of iodine in the soil, predisposing the population to iodine deficiency. New Zealand was the first country after Switzerland to introduce iodized salt to address widespread iodine deficiency, albeit at an initial low concentration of 5 mg I/kg; this was increased to 50 mg I/kg in 1939 [37]. Domestic household use of iodized salt, both at the table and in home cooking, successfully eradicated iodine deficiency in New Zealand by the early 1950s. Additional iodine was derived from dairy products when iodophors were used by the dairy industry from the 1960s to the 1980s. However, changes in food habits, including a reduction in the consumption of iodized salt in the home and a drop in the iodine content of dairy products when detergent-based sanitizers replaced iodophors, are factors believed to have contributed to the re-emergence of mild iodine deficiency in the 1990s [13]. Given the widespread nature of the deficiency, which was reported in all population groups, the most effective strategy identified by the government was mandatory fortification [41]. 

Dietary modeling was used to identify the best dietary approach to increase iodine intake without exceeding the upper limit of intake, with a particular focus on reducing the prevalence of iodine deficiency in pregnant women and children. In order to undertake this process, it was imperative that the iodine content of foods be included in the New Zealand Food Composition Database [108]. Because the most commonly eaten staple foods in the New Zealand diet are low in iodine, and the foods highest in iodine content are consumed in small quantities, a preliminary proposal was put forward to mandate the replacement of non-iodized salt with iodized salt in breads, breakfast cereals, and sweet biscuits (i.e., cookies). Because New Zealand imports and exports biscuits, a requirement to fortify biscuits with iodine would require separate production lines for both overseas and New Zealand biscuit producers. Furthermore, cereal manufacturers suggested that the application of a brine spray to breakfast cereals would produce inconsistent amounts of iodine in breakfast cereals. Thus, concerns about trade regulations for biscuits and technological concerns for breakfast cereal meant that bread, a staple food consumed by 87% of the New Zealand population, was chosen as the sole food for fortification; it was acknowledged that bread would not provide pregnant women with enough iodine to meet their higher requirements. A change to the Food Standards Code came into effect in September 2009 mandating the use of iodized salt in yeast-leavened bread; organic bread was exempt from this requirement [33]. The New Zealand Food Composition Database has included a revised iodine content for some breads, although more information on the iodine content of New Zealand breads can be found in a separate government report [109].

## 8. Discussion

Iodine has emerged as a nutrient of concern in many developed countries, in part because retail sales (and home use) of non-iodized salts may be increasing [11,110,111,112] and iodized salt is not always used in the commercially prepared foods that make up an ever-increasing component of the food supply yet whose consumption often leads to excessive sodium intake [11,110,111,112]. Also, some countries have not yet implemented mandatory salt iodization programs [51,112,113]. Pregnant women and young children are at highest risk of inadequate intake but other groups within populations may also be at risk. At present there is no simple or reliable way to assess the iodine status of an individual. Although other causes cannot be ruled out, altered thyroid function can suggest the presence of iodine deficiency. In the future, validated biomarkers of individual status (such as thyroglobulin levels) may become available [114]. In the meantime, despite the difficulty of directly assessing iodine status of individuals, practical diet-based approaches may prove useful. 

Individuals should be asked about the voluntary and involuntary factors that can affect iodine intake and status. This information can provide a basis for assessing risk and providing counseling. For example, interview methodology can be used to gather information on use of iodine-containing dietary supplements, and on intake of food sources of iodine such as dairy products and seafood, including fresh and saltwater fish and seaweeds. A growing concern in many countries is the adoption of dietary patterns that specifically exclude major sources of iodine, including low-salt [115], vegan [116,117,118], and Paleo diets [119]. Questionnaires must be attuned to phrasing; for example, it is important to enquire about use of dairy vs non-dairy milks, as the iodine content of milk alternatives often is very low [120]. In some situations, it may even be desirable to develop individualized advice regarding type of salt; for example, researchers in China are attempting to develop an online screening tool that informs consumers if they should consume iodized or non-iodized salt [121].

An additional topic that needs appropriate interview methodology includes the type of salt used in the home for cooking and at the table. For example, interviewers should ask how often new iodized salt is purchased, as the iodine content may decline over long periods of storage. Interviewers should also ascertain use of sea salt, which, if not fortified, may have a surprisingly low iodine content. Determining the amount of iodine coming from iodized cooking and table salt can be difficult to quantify for a number of reasons. Firstly, the interpretation of a sprinkle or pinch of salt will vary from person to person. Secondly, if food intake is being weighed, the weighing scales may not be sensitive enough to measure salt added at the table and weight recorded as null (i.e., 0) grams. Thirdly, when iodized salt is used for cooking vegetables or pasta, the amount of iodine in water that is discarded and the amount that becomes incorporated into the cooked food is unknown. Thus, iodine intakes determined using diet records or 24-h recalls are likely to underestimate actual iodine intake, particularly in individuals who regularly and generously add iodized salt to their food. A simpler approach is to add a set amount of iodine to anyone who reports use of iodized discretionary salt; in New Zealand, an additional 48 μg of iodine, representing the consumption of 1 g of salt (48 mg I/kg) per day, is included in the total daily iodine intake to account for discretionary use of iodized salt [109]. 

Data on the iodine content of foods can also be useful in research settings. Although seldom considered, an estimate of the iodine content of the diet determined using a validated iodine-specific food frequency questionnaire should be included as a variable in studies investigating thyroid function on disease and child development. The iodine content of the diet could also be used to stratify participants in randomized controlled trials of iodine supplementation. 

Another use of iodine databases is in the treatment of thyroid disease. Patients undergoing radioablation treatment for thyroid cancer are prescribed a several-week course of a low iodine diet, in order to enhance uptake of radioactive iodine [122,123]. The degree of success in reducing iodine intake usually is estimated using urinary iodine excretion [124]. Some patients find it difficult to adhere to these diets. Also, there is debate about the necessary degree of dietary restriction, as well as about the optimal time frame for dietary modification, both of which may depend, in part, on the background iodine content of the patient’s usual diet [125,126]. In some circumstances, iodine data from other countries has been used to develop dietary prescriptions for patients living in a different country [127]. Improved national-level databases will be useful in clinical practice and in research on low iodine diets.

Goitrogens are a chemically diverse group of compounds that have the capacity to interfere with uptake or utilization of iodine by the thyroid gland, and thus pose an additional source of dietary complexity important for understanding issues of adequacy of iodine intake [18]. The impact on thyroid status of eating these foods may depend on the quantity eaten and the background iodine content of the diet. High goitrogen intake may render marginal iodine intakes inadequate for physiologic demands and under some circumstances may actually contribute to goiter endemics and related disorders [128]. Dietary goitrogens often are inherent botanical constituents of foods; examples include cassava (cyanogenic glucosides), cruciferous vegetables (glucosinolates), and soy products (flavonoids). Dietary assessments should include information on intake of goitrogen-source foods, which can be very specific to geographic region, including cultural practices and economic issues related to the cost of foods. Special purpose databases or data tables with information on some of the goitrogenic constituents of foods (e.g., the USDA Flavonoid Database [74,129]) can provide useful corollaries for iodine composition tables and would have potential application for research and for counseling individuals at risk for thyroid disease. Environmental goitrogens also may present dietary exposures of concern in some situations and include perchlorates, nitrates, and disulfides. These compounds may derive from industrial contamination but may also occur naturally in soils and can subsequently leach into the water supply and thereby into foods [130]. Tobacco smoke presents another important goitrogenic exposure due to its thiocyanate content. As individuals are unlikely to be aware of ingestion of most environmental goitrogens, it may be useful to consider estimating exposure through the use of biomarkers [131,132].

In the US, the USDA Food Iodine Database project described in this paper complements the ODS-supported DSID and DSLD resources that provide data on the iodine content of supplements, necessary to determine total iodine intake. Once the USDA Food Iodine Database is complete, it will be possible to generate more complete estimates of iodine intake and iodine sources through linkage to dietary intake tools and to the data generated by surveys such as the NHANES, which heretofore has based its estimates of iodine intake on urinary iodine levels [133]. Also, the eventual availability of iodine values for a large number of foods with descriptive statistics that include multiple measures of variability (e.g., percentile cutoffs, coefficients of variation, and standard deviation) and central tendency (e.g., means and medians) will allow modeling of intakes that account for the spread of iodine levels in many types of foods, thus helping to develop appropriate methods for estimating intake of individuals and populations [58].

Another US concern, which also may be the case in other countries, is that information is available about the iodine content of infant formula but not breast milk. Databases on the typical iodine content of human milk would be desirable. Estimating the likely iodine intake of breast-fed infants is an additional key aspect of understanding adequacy of population level iodine status. 

## 9. Conclusions

Information about the iodine content of national food supplies is essential for understanding the state of human iodine nutrition around the world, with important applications in nutrition research, dietary counseling, treatment of thyroid disease, and public health practice. Collection and evaluation of dietary data are essential components of this work because there is no simple way at present to estimate iodine status of individuals. When possible, iodine composition databases for foods should be complemented by databases for dietary supplements in countries where supplements make a significant contribution to the total intake of iodine. Regularly updated composition data that reflect current food supplies are needed to support nutrition surveys, which generate critical data resources for understanding national-level concerns and identifying sub-populations at risk due to typical dietary patterns or increased physiological need. In future surveys, characterizing the relationship between iodine intake and thyroid function across populations and within population subgroups will require information on total intake, from all sources.

## Figures and Tables

**Table 1:** National food composition databases that include iodine.

Country	Year of Salt Iodization	URL	Database Name
Armenia	2004 [32]	pdf.usaid.gov/pdf_docs/Pdach758.pdf	Armenian Food Composition Table 2010
Australia	1953/54 ^1^ [33]	www.foodstandards.gov.au/science/monitoringnutrients/ausnut/foodnutrient/	AUSNUT 2011-13 Food Nutrient Database
Austria	1963 [34]	www.oenwt.at/content/naehrwert-suche/	OENWT Österreichische Nährwerttabelle
Bahrain	Not found ^2^	www.fao.org/fileadmin/templates/food_composition/documents/pdf/FOODCOMPOSITONTABLESFORBAHRAIN.pdf	Food Composition Tables for the Kingdome of Bahrain
Czech Republic	1950 [34]	www.nutridatabaze.cz/en/	Czech Food Composition Database
Denmark	1998 [34]	frida.fooddata.dk/AlpList.php	Danish Food Composition Databank
Finland	1949 [34]	fineli.fi/fineli/en/index	Fineli, National Food Composition Database in Finland
France	1952 [34]	pro.anses.fr/TableCIQUAL/	CIQUAL French Food Composition Table
Italy	1972 [35]	www.bda-ieo.it/wordpress/en/	Food Composition Database for Epidemiological Studies in Italy (Banca Dati di Composizione degli Alimenti per Studi Epidemiologici in Italia—BDA
Japan	No program	www.mext.go.jp/en/policy/science_technology/policy/title01/detail01/sdetail01/sdetail01/1385122.htm	Tables of Food Composition in Japan-2015-(7th Revised Ed)
Malaysia	2000 [36]	myfcd.moh.gov.my/	Malaysian Food Composition Database (MYFCD)
New Zealand ^3^	1924 [37]	www.foodcomposition.co.nz/concise-tables	Concise New Zealand Food Composition Tables 12th Ed
The Netherlands	1942 [34]	nevo-online.rivm.nl/ProductenZoeken.aspx	Dutch Nutrient Material File (NEVO)
Norway	1920 [34]	www.matvaretabellen.no/	Norwegian Food Composition Table
Poland	1997 [34]	www.izz.waw.pl/index.php?lang=en	Poland Food Composition Tables Database
Slovakia	1953 [34]	www.pbd-online.sk/	Slovak Food Composition Database Online
Slovenia ^4^	1964 [34]	opkp.si/en_GB/cms/vstopna-stran	OPKP (Open Platform for Clinical Nutrition)
Spain	1982 [35]	www.bedca.net/bdpub/index_en.php	Spanish Food Composition Database
Sweden	1936 [35]	www.livsmedelsverket.se/en/food-and-content/naringsamnen/livsmedelsdatabasen	Livsmedelsdatabasen—Swedish Food Composition Database
Switzerland	1922 [34]	www.naehrwertdaten.ch/	Swiss Food Composition Database
Tanzania	1990s [38]	www.hsph.harvard.edu/nutritionsource/food-tables/	Tanzania Food Composition Tables
Tunisia	1990s [39]	www.mpl.ird.fr/tahina/home/doc/sommaire_table_composition.pdf	Table de Composistion des Aliments Tunisiens
Turkey ^5^	1968 [34]	www.turkomp.gov.tr/?locale=en	Turkish Food Composition Database, TürKomp ^2^
UK	No program	www.gov.uk/government/publications/composition-of-foods-integrated-dataset-cofid	Composition of Foods Integrated Dataset (CoFID)

^1^ Iodized salt was only added to bread in 1953/1954 but was discontinued in the 1980s; iodized salt was available in Australia from this time. A 2009 iodization program applied to salt added to most bread [33,40]. ^2^ Search was conducted and salt iodization details were not located, but this does not confirm the absence of a program. ^3^ Database does not have updated iodine values for all fortified breads. ^4^ Database lacks some milk/dairy and beverage products. ^5^ Database iodine content is limited to table salt, fish, and shellfish.

**Table 2:** National food composition databases that do not include iodine.

Country	Year of Salt Iodization	URL	Source Name
Belgium	1990 [34]	www.nubel.com/fr/table-de-composition-des-aliments.html	Belgian Table of Food Composition
Brazil	No program	www.fcf.usp.br/tbca/	Brazilian Food Composition Table (TBCA)
Cameroon	Not found ^1^	www.academia.edu/5451699/A_review_of_composition_studies_of_Cameroon_traditional_dishes_Macronutrients_and_minerals	Journal Publication
Canada	1949 [41]	food-nutrition.canada.ca/cnf-fce/index-eng.jsp	Canadian Nutrient File (CNF)
Chile	1979 [42]	web.minsal.cl/composicion-de-alimentos/	Chilean Table of Chemical Composition of Foods, Update 2010
China	1995 [43]	www.neasiafoods.org/dataCenter.do?level=yycfk&language=us	Food and Nutrient Database, Food Nutrition Library
Costa Rica	1970 [44]	www.inciensa.sa.cr/actualidad/Tabla%20Composicion%20Alimentos.aspx	Tablas de Composición de Alimentos
Cuba	Not found ^1^	www.inha.sld.cu/	Tabla de Composición de Alimentos Utilizados en Cuba
Gambia	Not found ^1^	ilsirf.org/wp-content/uploads/sites/5/2017/03/Gambia2011FCT.pdf	Food Composition Table for Use in The Gambia
Germany	1959 [34]	www.blsdb.de/	German Nutrient Database—Bundeslebensmittelschlüssel
Greece	No program	www.hhf-greece.gr/tables/Home.aspx?l=en	Composition Tables of Foods and Greek Dishes
Iceland	No program	old.matis.is/english/service/product-development-and-entrepreneurship/nutrition/isgem-the-icelandic-food-composition-database/	ISGEM (The Icelandic Food Composition Database)
India	1983 [45]	ifct2017.com/wp-content/uploads/2017/05/ifct-doc.pdf	Indian Food Composition Tables
Ireland	No program	www.ucc.ie/archive/ifcdb/	Irish Food Composition Database
Israel	No program	www.health.gov.il/Subjects/FoodAndNutrition/Nutrition/professionals/Pages/Tzameret.aspx	Tzameret
Korea	No program	koreanfood.rda.go.kr/eng/fctFoodSrchEng/engMain	Korean Standard Food Composition Table
Lesotho	2000 [46]	ilsirf.org/wp-content/uploads/sites/5/2017/03/Lesotho2006FCT.pdf	Lesotho Food Composition Tables
Mexico	Not found ^1^	www.innsz.mx/2017/Tablas/index.html#page/8	Tablas de Composicion de Alimentos y Productos Alimenticios Mexicanos (Version condensada 2015)
Mozambique	2000 [47]	ilsirf.org/wp-content/uploads/sites/5/2017/03/Mozambique2011FCT.pdf	Food Composition Tables for Mozambique, Version 2
Nepal	1973 [48]	www.fao.org/fileadmin/templates/food_composition/documents/regional/Nepal_Food_Composition_table_2012.pdf	Food Composition Table for Nepal 2012
Papua New Guinea	1995 [49]	www.fao.org/docrep/007/y5432e/y5432e00.htm	The Pacific Islands Food Composition Tables
Portugal	No program	portfir.insa.pt/foodcomp/search	Portuguese Food Composition Table
Serbia	1937 [50]	www.serbianfood.info/lozinka1.php	Serbian Food & Nutrition Database
Singapore	1988 [51]	focos.hpb.gov.sg/eservices/ENCF/	Energy and Nutrient Composition of Food
South African	1995 [52]	safoods-apps.mrc.ac.za/foodcomposition/	South African Food Database System (SAFOODS)
Sweden	1936 [34]	www.livsmedelsverket.se/en/food-and-content/naringsamnen/livsmedelsdatabasen	Livsmedelsdatabasen—Swedish Food Composition Database
Thailand	1994 [53]	www.inmu.mahidol.ac.th/aseanfoods/download/books/dl1.php?file=A1	ASEAN Food Composition Tables
Togo	Not found ^1^	ilsirf.org/wp-content/uploads/sites/5/2017/03/TogoTable_de_Composition_des_Aliments.pdf	Table de Composition des Aliments du Togo
Uganda	1990s [54]	www.harvestplus.org/category/resource-type/technical-monographs	A Food Composition Table for Central and Eastern Uganda
Vietnam	1999 [55]	www.fao.org/fileadmin/templates/food_composition/documents/pdf/VTN_FCT_2007.pdf	Bảng Thành Phần Thực Phẩm Việt Nam Vietnamese Food Composition Table
United States	1924 [56]	ndb.nal.usda.gov/ndb/	USDA Food Composition Database

^1^ Search was conducted and salt iodization details were not located, but this does not confirm the absence of a program.

**Table 3:** Project Components for Developing the USDA Food Iodine Database.

Design phase (Completed) *Define research needs* *Review existing data* *Assess iodine distribution in the food supply* *Develop sampling design and calculate sample sizes*
Preliminary study phase (Completed) *Conduct stability studies* *Develop sample handling protocols* *Identify appropriate analytical methods and quality control materials*
Research implementation phase (Ongoing) *Conduct analyses and data quality control reviews* *Conduct ancillary studies* *Disseminate data and documentation*
